# Describing Deaths over a Decade: The Final Week of Life Among Hospitalized Children with Cancer

**DOI:** 10.3390/children13020218

**Published:** 2026-02-03

**Authors:** Meaghann S. Weaver, Jia Liang, Erica C. Kaye, Deena A. Levine, Cai Li, Andrea Heifner, Alejandra Gabela, Liza-Marie Johnson

**Affiliations:** 1 Bioethics Program, St Jude Children’s Research Hospital, 262 Danny Thomas Place, Memphis, TN 38105, USA; liza.johnson@stjude.org; 2 Division of Quality of Life and Palliative Care, Department of Oncology, St Jude Children’s Research Hospital, Memphis, TN 38105, USA; erica.kaye@stjude.org (E.C.K.); deena.levine@stjude.org (D.A.L.); 3 Department of Biostatistics, St Jude Children’s Research Hospital, Memphis, TN 38105, USA; jia.liang@stjude.org (J.L.); cai.li@stjude.org (C.L.); 4 Division of Critical Care Medicine, Department of Pediatric Medicine, St Jude Children’s Research Hospital, Memphis, TN 38105, USA; andrea.heifner@stjude.org (A.H.); alejandra.gabela@stjude.org (A.G.); 5 Department of Global Pediatric Medicine, St Jude Children’s Research Hospital, Memphis, TN 38105, USA; 6 Department of Hospitalist Medicine, St Jude Children’s Research Hospital, Memphis, TN 38105, USA

**Keywords:** pediatric oncology, pediatric cancer, end-of-life care

## Abstract

Background and Objectives: Little is known about the final week of life for inpatient pediatric oncology patients. The purpose of this study was to describe inpatient pediatric oncology deaths. Methods: Retrospective chart review of all patients who died in a large pediatric cancer center between 2007 and 2017. Demographic, diagnostic, and proximate cause of death information was extracted. Intensive care unit (ICU) admissions, chemotherapy receipt, medical interventions, and cardiopulmonary resuscitation (CPR) events one week, 48 h, and 24 h prior to death were obtained. Analysis included descriptive and statistical correlations. Results: 344 decedent pediatric oncology patients were included. Half of the patients died in the ICU (51%). The average age was 11.3 years (range 0.13–27.7 years). Most had ongoing evidence of disease (86%) with 20% receiving cure-directed chemotherapy during their final week. Receiving cure-directed chemotherapy was not associated with age, race, ethnicity, or diagnosis but was associated with a significantly increased likelihood of receiving CPR on the last day of life (*p* = 0.011). The majority (84%) of children did not receive CPR on their final day of life. Receipt of CPR was not associated with race/ethnicity. CPR was associated with younger age (*p* = 0.006), hematologic malignancies (*p* = 0.037), and ICU location (*p* < 0.001). Most patients were not on dialysis (84%), pressors (72%), or ventilated (60%) during the final 24 h of life. Compassionate extubation occurred in <6% of deaths. Conclusions: Most deaths in a pediatric cancer center occur in children with active disease. Continuation of cure-directed chemotherapy, age, diagnosis, and location of death has potential to influence end-of-life inpatient care.

## 1. Introduction

Cancer is the leading cause of death in children in the United States, after all injury-related causes [1]. Malignant neoplasms account for 9.1% of pediatric deaths nationwide [2]. The inpatient hospital setting represents the most common location of death for children with cancer [3,4]. A French population-based study found that 61% of patients aged 0–25 years who died in the hospital setting from cancer between 2014 and 2016 received high-intensity end-of-life care, including chemotherapy, within two weeks of death [5]. A retrospective decedent cohort of patients with childhood cancer who died between 2000 and 2012 in Ontario revealed that 41% received IV chemotherapy fourteen days prior to death [6]. A previous single institution study indicated that up to 20% of pediatric oncology patients receive maximal interventions until death [7].

Descriptive studies focusing on inpatient interventions for pediatric cancer in the final week of life are limited. Most of the existing research on end of life for children with cancer in the United States are based on palliative care cohort studies or are based on data restricted to specific locations, such as home-based hospice deaths [8,9]. The objective of this study was to describe the inpatient medical management of children with cancer during the last week of life, with a focus on intensive medical interventions, administration of chemotherapy, receipt of CPR, and location of care.

## 2. Materials and Methods

### 2.1. Population

All patients who died within any inpatient location at our institution between 2007 and 2017 were included. The single-study site is in Memphis, Tennessee (USA). This retrospective chart review study was deemed exempt from full institutional review board review due to a solely decedent cohort.

### 2.2. Data Extraction

The decade years were selected based on timing prior to merging to a new electronic health record system to ensure consistency in documentation of data points. General demographics (age, gender, race, ethnicity, religion, interpretive services use, and decision-maker in family unit) and diagnostic information (primary cancer diagnosis, prior resuscitation status, and transplant history) were recorded. Specific interventions were documented, such as ICU admissions; chemotherapy receipt; and medical interventions (pressors, ventilation, dialysis, antibiotics, artificial nutrition and hydration, chest tube, peritoneal drain, and surgery). Cardiopulmonary resuscitation (CPR) events before and at death were noted. Proximate cause of death was noted. The study team collected data from inpatient electronic health records that included up to four timepoints: terminal event, 24 h prior to the terminal event, 48 h prior to the terminal event, and 7 days prior to the terminal event. For patients who were not hospitalized at all prior to their terminal event, earlier timepoints had no corresponding inpatient data and were therefore labeled as “not applicable” (rather than “missing”). At each timepoint, the use of the medical intervention is recorded as new (no treatment in the previous timepoints but treated in the current timepoint), continued (treatment in previous and current timepoints), discontinued (treatments in both previous and current timepoints), and none (no treatment in both the previous and current timepoints).

### 2.3. Statistical Analysis

The Wilcoxon rank-sum test was used to test the difference in distribution between two groups of patients with continuous measurement, while the Kruskal-Wallis test was used to examine if there was at least one pair of group differences in three or more group comparisons. Chi-squared test was used to find the association between two categorical variables, while Fisher’s Exact test was used when there was a sub-category with less than 5 counts. Ordinary logistic regression was used to inspect multiple factors affecting the use of chemotherapy; chi-squared statistics and Wald-type confidence intervals were computed for each factor. All statistical analyses were performed based on a 95% level of confidence and using R statistical software (R Core Team 2024, version 4.4.2).

## 3. Results

A total of 344 decedent pediatric oncology patients were included in the study. The average age was 11.3 years (range 0.13–27.7 years). Demographics are provided in Table 1. The majority of patients had leukemia (42%) diagnosis, followed by solid tumor (29%), brain tumor (16%), lymphoma (8%), and other oncologic (5%) diagnoses. Half had previously undergone a bone marrow transplant (41% allogeneic and 7% autologous).

### 3.1. Medical Interventions

Medical interventions in the week, 48 h, and 24 h prior to death are depicted in Table 2. One-third (34%) of patients were ventilated during the final 24 h of life. Most patients were not on dialysis (84%) or pressors (72%) during the final 24 h of life. Few patients were newly initiated on pressors (10%), newly ventilated (6%), or started on dialysis (<1%) during the final 24 h of life. Pressor discontinuation occurred in the final 24 h of life for 3% of patients, ventilator discontinuation for 6%, and dialysis discontinuation for 5.5%. Compassionate extubation thus represents less than 6% of decedent deaths. Infectious-targeted agents (systemic antibiotics and/or antifungals) were continued in the final 24 h of life for 67% of patients. Over three-fourths of patients were on artificial nutrition and hydration during the final 24 h of life. Less than 12% of patients discontinued artificial nutrition and hydration in the seven-day period leading up to death.

### 3.2. Chemotherapy

In this cohort of children, 86% had evidence of active disease, with 20% receiving at least one form of disease-directed chemotherapy within the week prior to death. Receiving cure-directed chemotherapy within the week prior to death was not associated with age, race, ethnicity, or diagnosis across the three timepoints (one week, 48 h, and 24 h prior to death). Continuing cure-directed chemotherapy was associated with a significantly increased likelihood of receiving CPR on the last day of life (odds ratio = 3.175, CI 1.431–7.044; *p* = 0.011). Conversely, receiving palliative chemotherapy was associated with a significantly decreased likelihood of receiving CPR compared to receiving non-palliative chemotherapy (odds ratio = 0.056, CI 0.006–0.53, *p* = 0.006).

### 3.3. CPR

The majority (84%) of children did not receive CPR on their final day of life. An association was not found between receipt of CPR and race/ethnicity. We observed a significant difference (*p* = 0.006) in the age distribution, with those who did not undergo CPR at death being older (10.7 years, sd = 6.62) than those who did undergo CPR at death event (7.56 years, sd = 7.65). There was also significant difference in diagnosis and CPR on the final day of life, with a much lower proportion of patients with neuro-oncologic diagnoses and solid tumor undergoing CPR compared to patients with hematologic malignancies. (*p* = 0.037, see Table 3).

### 3.4. Location

The majority of patients (81%) had been admitted to the ICU sometime during their cancer treatment course. Half of the patients died in the ICU (51%) and half died on the inpatient floor (49%). Any lifetime admission to the ICU was associated with significantly increased likelihood of receiving CPR (odds ratio = 4.637, CI 1.40–15.36, *p* < 0.006). The ICU as a location of death was associated with a significantly increased likelihood of receiving CPR (odds ratio = 17.2, CI 6.06–48.88, *p* < 0.001).

### 3.5. Cause of Death

The majority of the proximal causes for death center around organ failure (63.1%), and oncologic or treatment sequela (20.5%). Proximal cause of death is strongly associated with the use of CPR 24 h before death (*p* = 0.0015), while it is not significant in 48 h and 7 days before death. A significantly higher percentage of patients underwent CPR 24 h before death in the organ failure, hemorrhage or cellular dysregulation, and infectious etiology groups compared to the oncologic or treatment sequela groups. At 48 h and 7 days before death, the majority of the patients did not undergo CPR.

## 4. Discussion

In this large series of children with cancer who died within a decade-long period, the majority of children had active disease and half died in the regular ward as compared to the ICU. Most patients did not receive cure-directed chemotherapy in their final week of life. Most patients did not receive CPR. Younger children and those with hematologic malignancies were more likely to receive CPR. Undergoing CPR was positively associated with being in the ICU on the last day of life. Notably, receiving palliative chemotherapy was associated with a significantly decreased likelihood of receiving CPR compared to receiving cure-directed chemotherapy. De-escalation of interventions and compassionate extubation were rare.

### 4.1. Use of Chemotherapy in Final Days

Interestingly, our study did not reveal demographic or diagnostic associations with receipt of chemotherapy at the end of life. Prozora and their team identified race and age as significantly associated with the use of chemotherapy in the last week of life [10]. Johnston et al. also reported higher intensity end-of-life care including chemotherapy in children from racially minoritized groups, with a bimodal distribution by age (higher intensity <5 years and 15–21 years) [4]. Multiple prior oncology studies have demonstrated that patients with hematologic malignancies are significantly more likely to receive curative-intent chemotherapy in their final days [10,11,12]. Notably, palliative care and strong interdisciplinary support from a well-resourced team (inclusive of chaplains, social workers, psychologists, etc.), many of whom families have known since time of diagnosis, may foster therapeutic relationships leading to deep communication, which can facilitate an understanding of the refractory nature of some cancer diagnoses versus a perception the team is “giving up” on a child when discontinuing chemotherapy regimens.

Intravenous chemotherapy and inpatient hospitalization are associated with reduced quality of life in children at the end of life [13]. Ongoing chemotherapy through the final days of death may not provide a meaningful benefit in terms of symptom control or survival, especially for patients with poor performance status. In adult cohorts, the negative effects of the continuation of disease-directed chemotherapy are frequently used as an indicator for potentially inappropriate care [4,14,15]. However, a study of parents of children whose cancer had no reasonable chance of being cured revealed that parents significantly favored continuation of chemotherapy (56%) compared with clinicians (16%; *p* < 0.0001) in the child’s final days of life [16]. This is likely a result of prognostic uncertainty and sustained hope for a cure [17,18]. Compassionate, clear communication that indicates when a child lacks a realistic chance of survival is helpful to parents in treatment decision making [19,20]. Palliative chemotherapy may serve as a comfort measure or life prolongation without undue burden, such that continuation may be aligned across goals [21]. Palliative chemotherapy may have a role in symptom relief at the end of life, although robust data is lacking [22].

### 4.2. CPR Cohort Considerations

Prior research from patients with a cancer diagnoses at our institution found no association between race and DNR status at the time of death (*p* = 0.57) [23]. A palliative-specific cohort study from our institution showed that Black patients are more likely to undergo CPR [24]. This study of all-comers with cancer did not reveal ethnic/racial differences in the likelihood of receipt of CPR. Our study did find an age association, with younger children being more likely to receive CPR. Across pediatric diagnoses and settings, infants and younger children are more likely than older children to receive CPR during inpatient deaths within and outside of ICUs [25,26,27]. The age finding may be a reflection of challenges with prognostication in younger children with potentially healthier vital organs. This may also reflect that cure-directed therapies have been attempted or even exhausted in older children (longevity of multiple disease-directed interventions). Conversely, older children may have more agency and verbal preference sharing to help transition family members toward accepting an end of life. Children with hematologic malignancies are more likely to receive CPR both in our study setting and in the existing literature [28,29]. This may be credited to the perceived reversible causes of arrest in hematologic malignancies: sepsis, tumor lysis, or bleeding [29,30]. In contrast, pediatric patients with advanced brain tumors more frequently experience progressive neurologic decline. Although CPR may be more common in children with hematologic malignancies, survival rates remain poor after resuscitation [31,32].

**Figure 1:**
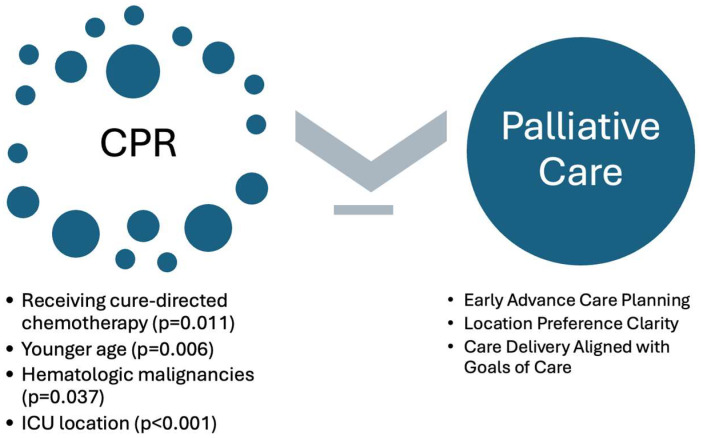
Variables relevant to receipt of CPR compared to palliative approach.

Palliative care and interdisciplinary team members should provide pediatric oncology families with younger children and/or with hematologic malignancies early opportunities to navigate advanced care planning (Figure 1). Revisiting the goals of care (time at home, specific milestones, symptom intervention preferences, protection from chaotic or crisis-based end-of-life interventions, etc.) can help transition goals into medical plans (location of care, symptom-first focus, maximizing home-based care models, and DNR status). In the terminal phase of cancer, care should be tailored to the unique needs and desires of the child and family, with the goal of providing the best possibility quality of life for the remaining days regardless of the location of care. Completion and update of inpatient code status and portable outpatient medical orders should include clear preferences regarding not only resuscitation preference but also location, such as potential preference for an inpatient comfort care room location near familiar nurses versus intensive care unit transfers. Simple language is profound: “We want to respect what matters most for your child. We want to ensure our care for your child matches what matters most to your family, including your preferred location of care. Could we review what today looks like and what you hope to protect and what you hope to avoid?” Teaching families what to expect (anticipatory guidance) can be empowering to foster a steady cadence in the setting of disease progression. Ideally, tangible support (equipment, hospice staffing, etc.) can be offered to ensure that home is a feasible care location. If the hospital is a preferred/likely care location, then families and the care team should work together to discuss comfort-first order sets, fostering a home-like feel to include visitation hour liberalization, and/or avoidance of overhead-codes or crisis response if the focus is comfort. Early introduction of flexible, creative, child-centric options in the context of therapeutic, trusted relationships, such as with palliative care team members, can be transformative in how a child’s end of life is experienced.

### 4.3. Location of Death—Intensive Care or Inpatient Floor

ICUs remain the most frequent location for pediatric cancer deaths in the United States. Not only do pediatric cancer patients comprise a higher percentage of ICU admissions, their mortality rates are far higher when compared to mortality rates for the non-cancer ICU pediatric population [33,34,35]. The patients who were ventilated at the time of death constituted more than half of the patients who died in the ICU setting during the study period. Most patients did not undergo de-escalation of biomedical interventions or receive compassionate extubation prior to death. As would be expected, children who experience mortality in an ICU experience a higher burden of intensive interventions at the end of life [36]. Parents of children in the intensive care unit are at risk for distress and post-traumatic stress, both during admission and into bereavement [37,38,39].

Our study found a more even proportion of deaths occurring in the non-ICU inpatient setting compared to prior multicenter studies, which reported that <40 to <15% of pediatric deaths occur in the general hospital ward. This may be related to the level of care our inpatient staff/rooms are equipped to handle to accommodate acuity. Many of our patients maintain years of longitudinal relationships with their disease-specific inpatient floor team and prefer to remain in that familiar setting for end of life. Prior work from the study team members revealed that lower odds of dying in the ICU in our setting were found in patients with hospice involvement (OR, 0.02; *p* < 0.0001) and documentation of advance directives at the time of death (OR, 0.37; *p* = 0.033) [40]. Access to hospice services remains disparate with hospice deserts across the United States, [41] making home or hospice location deaths impossible for many pediatric cancer patients receiving care in our setting due to national and international catchment [42].

### 4.4. Limitations

Our findings should be contextualized to the culture and variations that exist in a single-site cancer research referral site. Potentially meaningful data points and variables were not included in the study (such as relapse or recurrence status and caregiver demographics). We did not assess subspeciality palliative care’s impact because the study team has previously published that data [43,44]. We only included inpatients and therefore our findings are exclusive of patients who may have died at home. Despite these recognized limitations, this study on the location of death in pediatric oncology is important because different care settings (inpatient, ICU, and urgent care) reflect different goals and levels of medical intervention. The findings can help to inform potential palliative care access points, grief and resiliency staff resource allocation, and staff training specific to end-of-life bedside care.

## 5. Conclusions

This study provided decade-long descriptive data summarizing end-of-life care for hospitalized children from a large academic cancer center. The findings can impact future clinical care relevant to the interplay between factors such as curative-directed therapy and care location (ICU) and experiences in the final week of life. Clinically, the findings offer a relevant reminder of the importance of early advance care planning and compassionate discussions regarding preferences for the location of end-of-life care. Further research is warranted to learn about the impact of medical interventions, chemotherapy continuation, location of inpatient death, and CPR on families and healthcare providers.

## Figures and Tables

**Table 1:** Demographics (n = 344).

Variable	Result
0–7 years	126 (36.63%)
8–13 years	79 (22.97%)
14–18 years	72 (20.93%)
>18 years	67 (19.48%)
Gender	
Male	205 (59.6%)
Female	139 (40.4%)
Race	
White	223 (67.5%)
Black/African American	85 (24.6%)
Other	14 (4.1%)
Asian	6 (1.7%)
Unknown	1 (0.3%)
Ethnicity	
Non-Hispanic	267 (77.6%)
Hispanic/Latino	70 (20.3%)
Unknown	7 (2%)
Religion	
Christian	270 (78.5%)
Unknown/Unaffiliated	60 (17.4%)
Muslim	6 (1.7%)
Amish	3 (0.9%)
Hindu	2 (0.6%)
Other	2 (0.6%)
Jewish	1 (0.3%)
Location	
ICU	174 (50.6%)
Inpatient Unit	170 (49.4%)
Interpreter Services	
No	286 (83.1%)
Yes	58 (16.9%)
Decision Maker	
Both parents	190 (55.2%)
Mom	84 (24.4%)
Self with Parent Actively (loss of capacity)	32 (9.3%)
Other	16 (4.7%)
Dad	10 (2.9%)
Self	12 (3.5%)
Primary Cancer Diagnosis	
Hematologic malignancies	172 (50.0%)
Solid Tumor	99 (28.8%)
Brain Tumor	56 (16.3%)
Bone Marrow Transplant	17 (4.9%)
Ever in PICU	
Yes	280 (81.4%)
No	64 (18.6%)
Previous Resuscitation (Code Blue Event)	
No	275 (79.9%)
Yes	69 (20.1%)
Bone Marrow Transplant History	
No	179 (52.0%)
Yes (allogeneic)	142 (41.3%)
Yes (autologous)	23 (6.7%)
Evidence of Disease 7 days before death	
Yes	294 (85.5%)
No	50 (14.5%)

**Table 2:** Medical interventions the week before death (n = 344).

Variable	7 Days Before Death	48 h Before Death	24 h Before Death
Interventions the week before death			
PICU Setting			
Yes	131 (38.1%)	156 (45.3%)	176 (51.2%)
No	213 (61.9%)	188 (54.7%)	168 (48.8%)
CPR			
Yes	1 (0.3%)	1 (0.3%)	55 (16%)
No	343 (99.7%)	343 (99.7%)	289 (84%)
Pressor Use			
New	22 (6.4%)	15 (4.4%)	34 (9.9%)
Continued	28 (8.1%)	48 (14%)	53 (15.4%)
Discontinued	12 (3.5%)	4 (1.2%)	10 (2.9%)
None *	282 (82%)	277 (80.5%)	247 (71.8%)
Ventilator Use			
New	22 (6.4%)	10 (2.9%)	22 (6.4%)
Continued	84 (24.4%)	106 (30.8%)	96 (27.9%)
Discontinued	8 (2.3%)	0 (0%)	21 (6.1%)
None *	230 (66.9%)	228 (66.3%)	205 (59.6%)
Dialysis			
New	15 (4.4%)	4 (1.2%)	3 (0.9%)
Continued	41 (11.9%)	46 (13.4%)	34 (9.9%)
Discontinued	4 (1.2%)	7 (2%)	19 (5.5%)
None *	284 (82.6%)	287 (83.4%)	288 (83.7%)
Antibiotics or antifungals			
New	18 (5.2%)	4 (1.2%)	1 (0.3%)
Continued	254 (73.8%)	252 (73.3%)	230 (66.9%)
Discontinued	19 (5.5%)	16 (4.7%)	27 (7.8%)
None *	53 (15.4%)	72 (20.9%)	86 (25%)
Artificial Nutrition and Hydration			
New	39 (11.3%)	12 (3.5%)	8 (2.3%)
Continued	241 (70.1%)	267 (77.6%)	258 (75%)
Discontinued	7 (2%)	9 (2.6%)	23 (6.7%)
None *	57 (16.6%)	56 (16.3%)	55 (16%)
Disease-Directed Chemotherapy			
New	8 (2.3%)	4 (1.2%)	0 (0%)
Continued	51 (14.8%)	45 (13.1%)	33 (9.6%)
Discontinued	20 (5.8%)	10 (2.9%)	17 (4.9%)
None *	265 (77%)	285 (82.8%)	294 (85.5%)
Palliative Chemotherapy			
Unknown	0 (0%)	4 (8.2%)	2 (6.1%)
Yes	19 (32.8%)	17 (34.7%)	15 (45.5%)
No	39 (67.2%)	28 (57.1%)	16 (48.5%)
Chest Tube			
New	9 (2.6%)	3 (0.9%)	1 (0.3%)
Continued	20 (5.8%)	29 (8.5%)	27 (7.8%)
Discontinued	1 (0.3%)	0 (0%)	3 (0.9%)
None *	314 (91.3%)	311 (90.7%)	313 (91%)
Peritoneal Drain			
New	2 (0.6%)	2 (0.6%)	2 (0.6%)
Continued	8 (2.3%)	11 (3.2%)	11 (3.2%)
Discontinued	1 (0.3%)	0 (0%)	0 (0%)
None *	333 (96.8%)	331 (96.2%)	331 (96.2%)
Surgery			
Unknown/Missing	0 (0%)	0 (0%)	1 (0.3%)
Yes	16 (4.7%)	4 (1.2%)	8 (2.3%)
No	328 (95.3%)	340 (98.8%)	335 (97.4%)

Table 2 terminology: new means no treatment in the previous timepoints but treated in the current timepoint; continued means treatment in previous and current timepoints; discontinued means treatments in both previous and current timepoints; and none means no treatment in both the previous and current timepoints. * Some of the patients were not hospitalized at this timepoint and thus “none” also includes patients not hospitalized and thus not on a given intervention in the inpatient setting.

**Table 3:** Cardiopulmonary resuscitation on the final day of life per diagnostic sub-cohorts (n = 298).

	No CPR (N = 266, 89.3%)	Yes CPR (N = 32, 10.7%)	*p*-Value
Age			0.0108 ^1^
Mean (SD)	10.7 (6.62)	7.56 (7.65)	
Diagnosis			
Leukemia	121 (45.5%)	22 (68.8%)	0.037 ^2^
Solid Tumor	91 (34.2%)	8 (25.0%)	
Brain Tumor	54 (20.3%)	2 (6.3%)	

^1^ Wilcoxon rank-sum test; ^2^ Fisher’s Exact test.

## Data Availability

De-identified, aggregated data available upon request from the corresponding author.

## Abbreviations

The following abbreviations are used in this manuscript:

CPR	Cardiopulmonary Resuscitation
DNR	Do Not Resuscitate
ICU	Intensive Care Unit
